# Discovery of Potent EGFR Inhibitors for Del19 and L858R/T790M Mutants Through Structure‐Based Virtual Screening and Biological Evaluation

**DOI:** 10.1111/cbdd.70391

**Published:** 2026-09-11

**Authors:** Ting Xu, Yuchen Zhao, Mengfei Chen, Lijuan Zuo, Hanshuang Cai, Yuguo Liu, Chuanfeng Liu, Jing Shi

**Affiliations:** ^1^ Department of Pharmacy (Shandong Provincial Key Traditional Chinese Medical Discipline of Clinical Chinese Pharmacy), Shandong Cancer Hospital and Institute Shandong First Medical University and Shandong Academy of Medical Sciences Jinan People's Republic of China; ^2^ Taian Central Hospital General ICU Zone 2 Taian China

**Keywords:** drug resistance, EGFR inhibitor, EGFR^Del19^, EGFR^L858R/T790M^, structure‐based virtual screening

## Abstract

Epidermal growth factor receptor (EGFR) mutation‐mediated drug resistance represents a major clinical challenge in the treatment of non‐small cell lung cancer (NSCLC). In this study, the lead compound **XJ2‐2** was identified through structure‐based virtual screening. It exhibited potent anti‐proliferative activity against both EGFR^Del19^ mutant PC‐9 cells and EGFR^L858R/T790M^ mutant H1975 cells, with IC_50_ values of 0.88 ± 0.12 μM and 0.77 ± 0.09 μM, respectively. Mechanistic studies have demonstrated that **XJ2‐2** directly targets and binds to the EGFR, thereby effectively inhibiting EGFR phosphorylation and its downstream signaling pathways in both in vitro and in vivo models. In tumor‐bearing mouse models, **XJ2‐2** significantly reduced tumor burden. Preclinical safety evaluation indicated that no treatment‐related toxicity was observed for **XJ2‐2** at all tested doses. With its novel molecular scaffold and mutant‐selective inhibitory profile, **XJ2‐2** represents a promising lead compound for overcoming NSCLC drug resistance and advancing the development of next‐generation EGFR inhibitors.

## Graphical abstract

XJ2‐2 overcomes NSCLC by blocking EGFR‑driven AKT/ERK cascades.

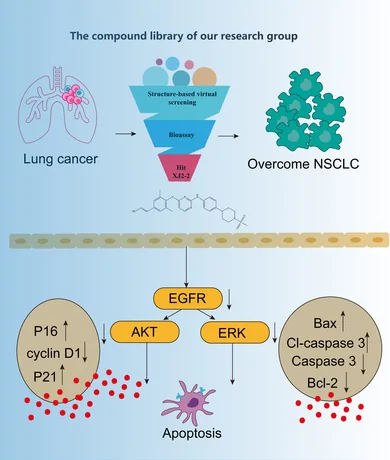

## 1 Introduction

Non‐small cell lung cancer (NSCLC) is the leading cause of mortality associated with solid tumors worldwide (DeSantis et al. 2017; Shepherd et al. 2005; Leiter et al. 2023). The epidermal growth factor receptor (EGFR) has emerged as a critical therapeutic target in its management (Sharma et al. 2007; Avraham and Yarden 2011; Gazdar 2009; Rosell et al. 2010). Currently, several small molecule inhibitors targeting the EGFR tyrosine kinase (TKI) have been approved by the FDA for clinical application (Niggenaber et al. 2020). The first‐generation reversible EGFR TKIs, such as gefitinib, exert their effects by competitively occupying the ATP‐binding pocket, thereby inhibiting autophosphorylation of the EGFR kinase domain and its downstream signaling pathways (Maemondo et al. 2010; Paz‐Ares et al. 2017). These agents have demonstrated significant clinical efficacy and have prolonged progression‐free survival (PFS) in patients. However, their reversible binding mechanism often leads to the development of acquired resistance, with the T790M “gatekeeper” mutation being the most prevalent resistance mechanism (Westover et al. 2018; Yun et al. 2008). To address this limitation, second‐generation (e.g., afatinib) and third‐generation (e.g., osimertinib) irreversible EGFR TKIs were developed (Cho et al. 2023; Remon et al. 2018). These compounds covalently and persistently bind to EGFR and other ErbB family members, effectively overcoming T790M‐mediated resistance and extending first‐line treatment PFS to 11–14.7 months (Mok et al. 2017; Cooper et al. 2022). Despite these advancements, their broad kinase inhibition profiles are associated with increased toxicity (Tan et al. 2018; Tsuboi et al. 2023). Moreover, resistance mechanisms continue to evolve, including the C797S mutation that prevents covalent binding, activation of bypass signaling pathways, and tumor phenotypic transformation (Piotrowska et al. 2018; Thress et al. 2015; Wheeler et al. 2010; Chen et al. 2018; Gao et al. 2025), all of which significantly compromise long‐term therapeutic efficacy (Patel et al. 2017; He et al. 2021; Ahmad and Patel 2025). Therefore, there is an urgent need for the development of next‐generation EGFR inhibitors based on novel molecular scaffolds to effectively address the challenges posed by multi‐drug resistance.

Structure‐based virtual screening (SBVS), due to its capacity to efficiently identify novel molecular scaffolds, has emerged as a crucial strategy in the discovery of lead compounds within the field of computer‐aided drug design (Meng et al. 2011; Woo et al. 2023; Forli et al. 2016; Friesner et al. 2004; Halgren et al. 2004; Sabe et al. 2021). In this study, SBVS was employed to perform a large‐scale screening of a compound library containing 4000 molecules from our research group, with the objective of targeting key drug‐resistant mutants of the EGFR. Four lead molecules featuring distinct structural scaffolds were successfully identified. The anti‐proliferative activities of these compounds were evaluated against EGFR^Del19^ mutant PC‐9 cells, EGFR^L858R/T790M^ double mutant H1975 cells, and human normal lung epithelial BESA‐2B cells using the CCK‐8 assay. The results revealed that compound **XJ2‐2** exhibited potent anti‐proliferative activity against both mutant cell lines, with IC_50_ values of 0.88 ± 0.12 μM for PC‐9 cells (EGFR^Del19^) and 0.77 ± 0.09 μM for H1975 cells (EGFR^L858R/T790M^). Based on these findings, comprehensive investigations including target validation, in vitro and in vivo mechanism‐of‐action studies, and in vivo pharmacodynamic evaluations of **XJ2‐2** were carried out. The accumulated experimental evidence consistently confirmed that **XJ2‐2** is a potent lead compound that specifically targets EGFR and exhibits robust anti‐tumor activity. This study not only identified a novel chemical scaffold with potential for overcoming acquired resistance in NSCLC, but also provided a promising candidate molecule for the development of next‐generation EGFR allosteric inhibitors.

## 2 Results and Discussion

### 2.1 Receptor‐Based Virtual Screening

**FIGURE 1:**
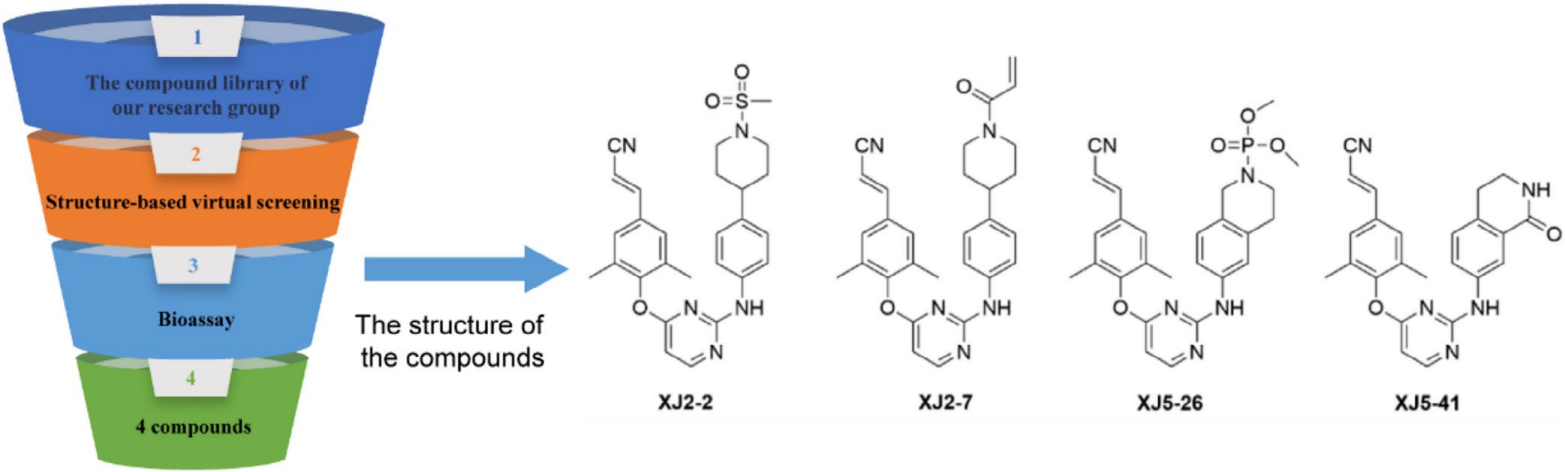
The structure of the compounds.

This study implemented a receptor‐based virtual screening strategy. Initially, the compound library from our research group was preprocessed. This preprocessing included adjustment of protonation states (pH 7.0 ± 2.0), generation of chiral isomers, and energy minimization using the OPLS4 force field. Subsequently, a preliminary drug‐likeness screening based on Lipinski's Rule of Five was performed to enrich the library with compounds possessing favorable pharmacokinetic properties. The crystal structure of the target protein (PDB ID: 2JIU) was prepared using the Protein Preparation Wizard, which involved hydrogen bond network refinement, adjustment of protonation states at physiological pH (7.0), and energy optimization with the OPLS 4 force field. Following the definition of the binding site based on the co‐crystallized ligand, a docking grid was generated. Hierarchical virtual screening was then conducted using the Glide module (v7.4). The first stage involved high‐throughput virtual screening (HTVS), retaining the top 1% of compounds based on docking scores. These were further refined through standard precision (SP) docking, followed by extra precision (XP) docking of the top 10% compounds. Finally, the top 50% of compounds based on XP scores were retained for further analysis. Structure‐based molecular interaction analysis was performed on the XP results, focusing on the evaluation of inhibitory interaction patterns between the compounds and key active‐site residues (e.g., Asn123 and Asp189), including hydrogen bonds and π–π stacking interactions, which were consistent with previously reported findings. Compounds meeting the interaction criteria along with their corresponding XP Glide G scores (kcal/mol) are summarized in Table 1, and their chemical structures are presented in Figure 1. Ultimately, 4 lead compounds were selected for experimental validation based on dual criteria: optimal Glide G scores and the presence of a complete network of key interactions.

**TABLE 1:** The Glide XP score and molecular weight (MW) of the compound.

Compound	G‐Score (kcal/mol)	MW^a^
**XJ5‐41**	−7.865	411.17
**XJ2‐2**	−7.847	503.2
**XJ2‐7**	−6.128	479.23
**XJ5‐26**	−6.089	505.19

^a^ Molecular weight (MW) of the molecule (range or recommended values: 130.0–725.0).

To evaluate the reliability of the molecular docking protocol, we employed a redocking strategy by re‐docking the co‐crystallized native ligand into its corresponding binding site and calculating the root‐mean‐square deviation (RMSD) between the obtained docked conformation and the crystallographic reference conformation. As shown in Figure S5, the RMSD value was 1.714 Å, significantly lower than the commonly accepted empirical threshold of 2.0 Å, indicating that the docking protocol accurately reproduces the experimental binding mode of the ligand. This result fully validates the suitability of the scoring function and search algorithm, thereby providing a reliable methodological foundation for subsequent structure‐based virtual screening studies.

### 2.2 Evaluation of the Anti‐Lung Cancer Cell Activity and Mutant EGFR Inhibitory Effects of Compounds

To evaluate the anti‐tumor activity of the target compound, we determined the half‐maximal inhibitory concentration (IC₅₀) of a series of compounds against the proliferation of two EGFR‐mutated non‐small cell lung cancer cell lines—PC‐9 (EGFR^Del19^) and H1975 (EGFR^L858R/T790M^)—and assessed their cytotoxicity, expressed as CC₅₀, in the normal human lung epithelial cell line BEAS‐2B. Osimertinib was used as the positive control. As shown in Table 2, all tested compounds exhibited anti‐proliferative effects. Notably, compound **XJ2‐2** demonstrated the most potent inhibitory activity, with IC₅₀ values of 0.88 ± 0.12 μM in PC‐9 cells and 0.77 ± 0.09 μM in H1975 cells, respectively. The CC₅₀ value in BEAS‐2B cells was 7.21 ± 0.34 μM, yielding selectivity indices (SI = CC₅₀/IC₅₀) of 8.2 for PC‐9 and 9.4 for H1975. These results indicate that **XJ2‐2** exhibits strong anti‐proliferative activity against EGFR‐mutant cancer cells with relatively low cytotoxicity toward normal cells.

**TABLE 2:** The antiproliferative activity of selected compounds.

Compounds	Antiproliferative activity @ IC_50_(μM)^a^				
	PC‐9 (EGFR^Del19^)	H1975 (EGFR^L858R/T790^)	BESA‐2B	SI^b^	SI^c^
**XJ2‐2**	0.88 ± 0.12	0.77 ± 0.09	7.21 ± 0.34	8.2	9.4
**XJ2‐7**	1.86 ± 0.32	5.12 ± 1.12	7.90 ± 0.78	4.24	1.54
**XJ5‐26**	4.95 ± 0.87	8.04 ± 1.01	4.23 ± 0.86	0.85	0.52
**XJ5‐41**	3.63 ± 0.76	9.62 ± 2.43	> 10	2.76	1.03
**Osimertinib**	0.53 ± 0.06	5.43 ± 0.42	5.26 ± 0.98	9.92	0.96

^a^ Values are average of three independent determinations. Data are presented as mean ± SD.

^b^ Selectivity Index (SI): PC‐9 (EGFR^Del19^) IC_50_/BESA‐2B IC_50_ value.

^c^ Selectivity Index (SI): H19759 (EGFR^L858R/T790^) IC_50_/BESA‐2B IC_50_ value.

### 2.3 Identification of the Target Protein of Compound XJ2‐2

**FIGURE 2:**
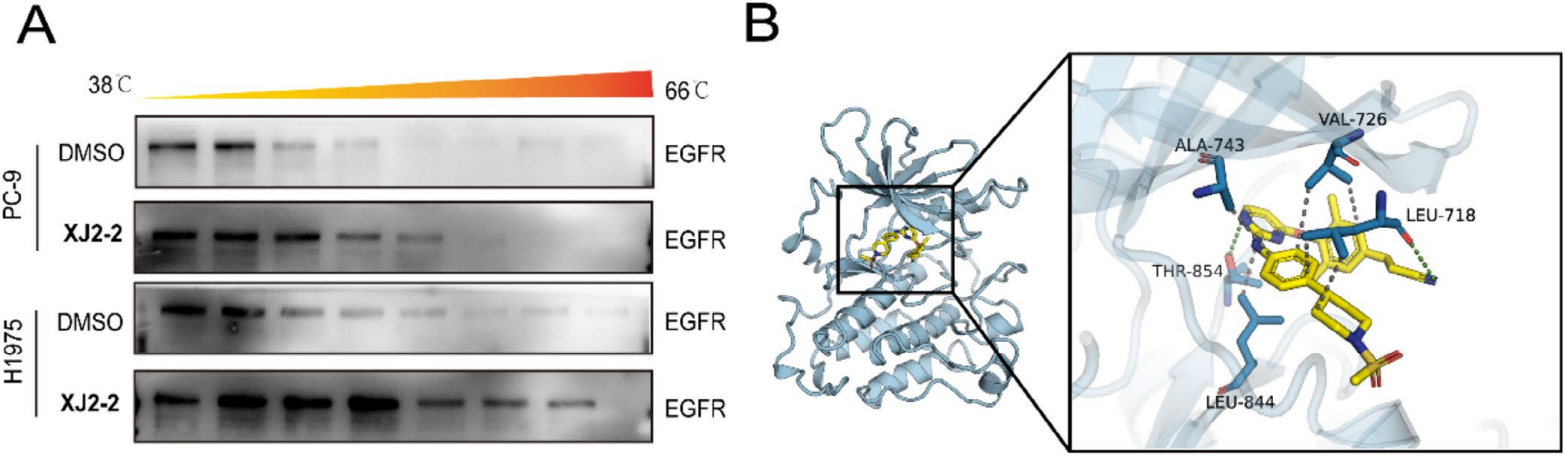
(A) Thermal stability of EGFR was evaluated by CETSA analysis in PC‐9 and H1975 cells treated with **XJ2‐2** (10 μM). (B) Binding interaction of the representative **XJ2‐2** with EGFR (PDB ID: 2JIU). In panels, blue dashed lines denote hydrogen bonds, gray dashed lines represent hydrophobic interactions, and orange dashed lines indicate π–π stacking interactions.

To confirm that compound **XJ2‐2** directly targets and interacts with the EGFR, we employed the cellular thermal shift assay for validation. As shown in Figure 2A, in PC‐9 and H1975 cells treated with **XJ2‐2**, the degradation rate of EGFR protein was significantly reduced with increasing temperature compared to the DMSO control group, indicating enhanced thermal stability. This thermal shift phenomenon directly demonstrates that **XJ2‐2** specifically binds to the EGFR protein within cells and confers resistance to heat‐induced degradation by stabilizing its conformation, thereby providing direct evidence of target engagement.

Based on this, we conducted molecular docking simulations to predict the potential binding interaction between compound **XJ2‐2** and the EGFR. The results revealed that **XJ2‐2** was deeply embedded within the ATP‐binding pocket of the EGFR kinase domain, forming a synergistic multi‐mechanism interaction network (Figure 2B). Specifically, the ligand **XJ2‐2** establishes multiple stable hydrophobic interactions and hydrogen bonds with EGFR. Specifically, hydrophobic contacts are observed with ALA‐743, VAL‐726, LEU‐844, and LEU‐718, while hydrogen bonding is primarily mediated by THR‐854 and LEU‐718, both of which significantly enhance the binding stability between the ligand and the receptor. In conclusion, **XJ2‐2** achieves high‐affinity binding to EGFR through multivalent non‐covalent interactions, providing a structural basis for further investigations.

### 2.4 Molecular Dynamics Simulation Characterization of the Interaction Between EGFR and Compound XJ2‐2

**FIGURE 3:**
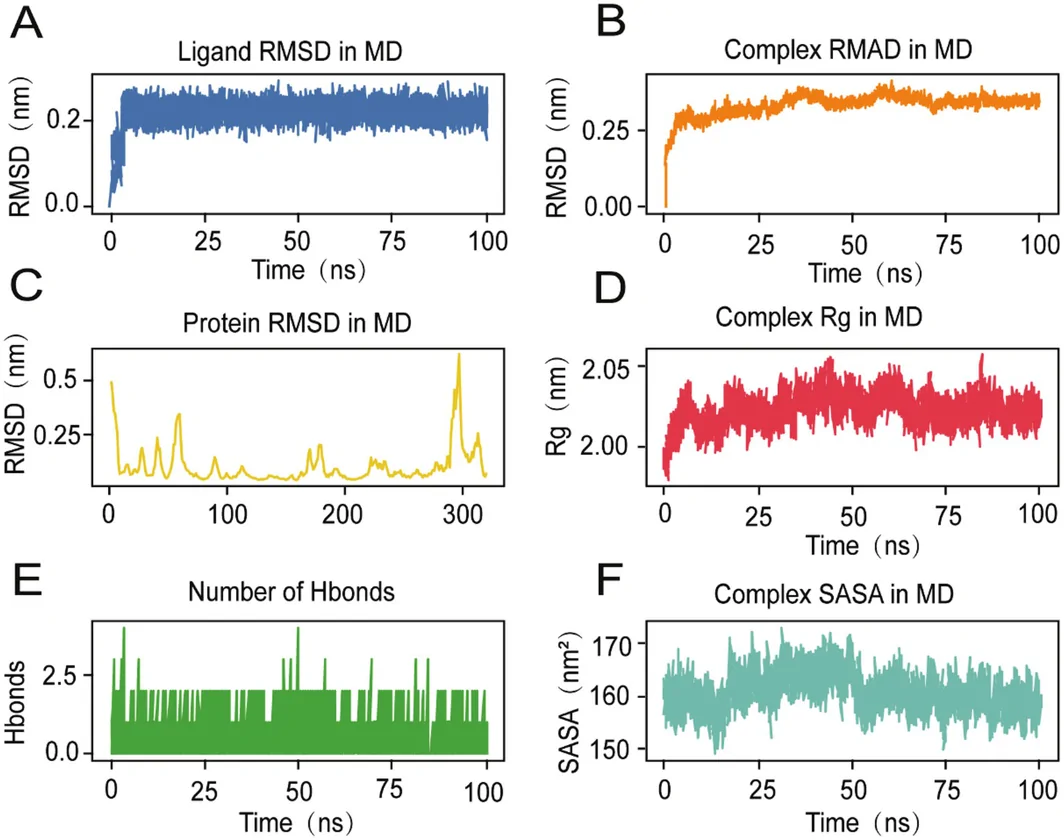
This figure illustrates the time‐dependent variations of key parameters during molecular dynamics (MD) simulations: (A) root mean square deviation (RMSD) of the ligand, (B) RMSD of the protein–ligand complex, (C) root mean square fluctuation (RMSF) of the protein, (D) radius of gyration (Rg) of the complex, (E) number of hydrogen bonds, and (F) solvent accessible surface area (SASA).

Based on the initial structure of the **XJ2‐2**/EGFR complex derived from molecular docking, this study performed all‐atom molecular dynamics simulations using the AMBER 24 software package. In molecular dynamics simulations, the root mean square deviation (RMSD) of the ligand initially increases rapidly before transitioning into a stable phase with minimal fluctuations, indicating rapid conformational adaptation and maintenance of a consistent binding pose without significant drift or repositioning (Figure 3A). Similarly, the RMSD of the protein‐ligand complex rises initially and subsequently stabilizes within a narrow fluctuation range, suggesting that the overall structure achieves a dynamic equilibrium after energy minimization, with no large‐scale conformational rearrangements—thereby preserving structural integrity and stability (Figure 3B). The root mean square fluctuation (RMSF) profile of the protein reveals low backbone residue fluctuations across most regions, with elevated values only at terminal segments and flexible loops, while key binding site residues exhibit minimal mobility, underscoring the rigidity of the binding pocket and its role in maintaining consistent binding interactions and structural stability (Figure 3C). The radius of gyration (Rg) of the complex remains consistently stable throughout the simulation, exhibiting only minor variations, which indicates a compact and well‐maintained overall architecture without notable expansion or collapse (Figure 3D). Furthermore, the number of hydrogen bonds, although subject to continuous dynamic exchange, remains within a stable range, reflecting a persistent and functionally relevant hydrogen bond network that contributes to binding stability (Figure 3E). Finally, the solvent accessible surface area (SASA) displays moderate and steady fluctuations without directional trends, indicating a stable level of protein surface exposure and absence of unfolding or compression events, thus highlighting the system's inherent capacity to preserve structural integrity in an aqueous environment (Figure 3F).

### 2.5 The EGFR Inhibitor XJ2‐2 Effectively Inhibits EGFR Signaling in Cancer Cells Harboring the Del19 and L858R/T790M Mutations

**FIGURE 4:**
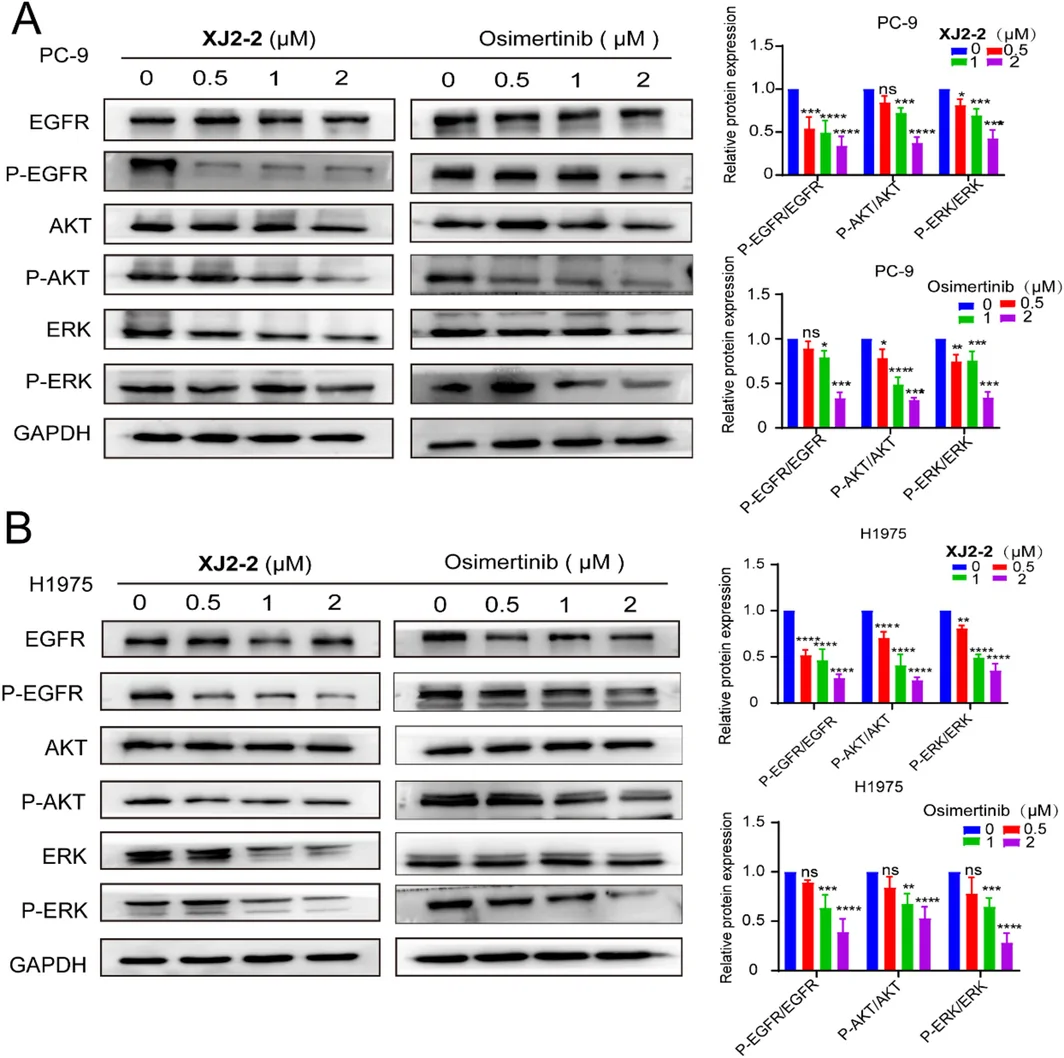
**XJ2‐2** inhibits the EGFR signaling pathway in vitro. (A) Cells were treated with specified concentrations of **XJ2‐2** and osimertinib for 24 h. Subsequently, cell proteins were extracted, and the levels of P‐EGFR, P‐AKT, and P‐ERK were detected by immunoblotting. (B) Quantitative analysis of protein expression levels was performed. The representative data presented are derived from at least three independent experiments. Compared with the control group, statistical significance is denoted as follows: *p < 0.05; **p < 0.01; ***p < 0.001; ****p < 0.0001.

PI3K/AKT is a canonical downstream signaling pathway of EGFR. Aberrant activation of this pathway can modulate cellular processes such as tumor cell invasion, migration, apoptosis, and cycle regulation, thereby contributing to tumor initiation and progression. Based on this regulatory mechanism, the present study employed the third‐generation EGFR inhibitor osimertinib as a positive control to systematically evaluate the inhibitory effect of compound **XJ2‐2** on the EGFR signaling axis in PC‐9 and H1975 cell lines using WB analysis (Figure 4A,B). The experimental results demonstrated that **XJ2‐2** significantly suppressed EGFR autophosphorylation in a concentration‐dependent manner and effectively inhibited the activation of its downstream effectors AKT and ERK1/2, with an inhibitory potency comparable to that of osimertinib. The concurrent downregulation of these phosphorylation events confirms that **XJ2‐2** specifically targets and inhibits the kinase activity of mutant EGFR, thereby disrupting the PI3K/AKT‐MAPK signaling pathway, underscoring its precise and effective targeting capability in EGFR‐driven tumor models.

### 2.6 Compound XJ2‐2 Inhibits Tumor Cell Colony Formation, Migration, and Invasive Capacity

**FIGURE 5:**
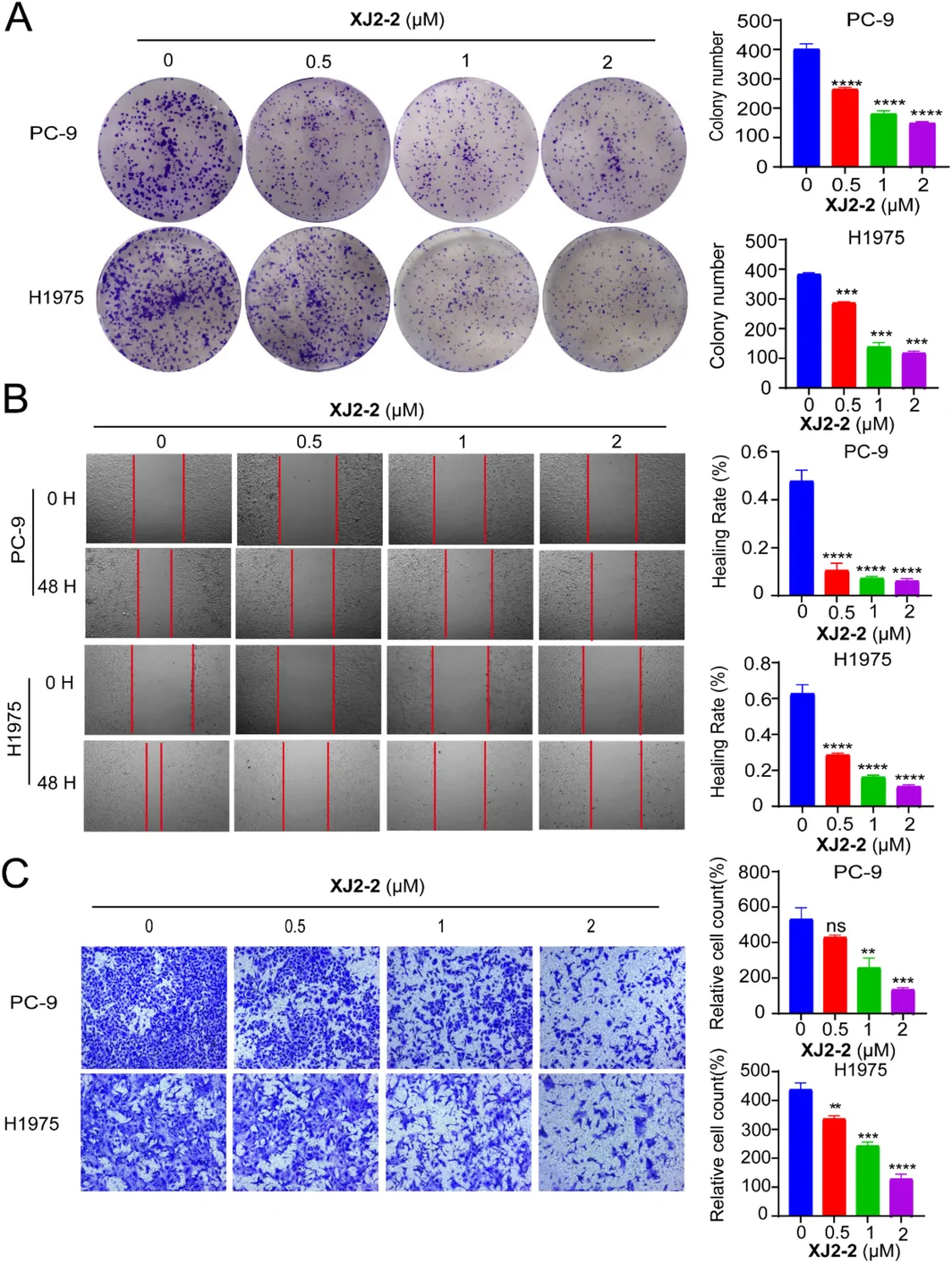
**XJ2‐2** inhibits the migration and invasion of NSCLC cells. (A) The proliferative capacity of PC‐9 and H1975 cells was assessed via colony formation assays, with the number of colonies quantitatively analyzed using ImageJ software. (B) The migratory capacity of PC‐9 and H1975 cells was evaluated through wound healing assays, and the migration rate was quantified using ImageJ software. (C) The invasive capacity of PC‐9 and H1975 cells was determined by transwell invasion assays, with the number of invasive cells quantitatively analyzed using ImageJ software. Data are expressed as mean ± SD (*n* = 3). Images from wound healing and invasion assays were acquired using an inverted fluorescence microscope. Statistical significance compared to the control group: *p < 0.05; **p < 0.01; ***p < 0.001; ****p < 0.0001.

Aberrant activation of the EGFR is closely associated with the proliferation and metastasis of tumor cells. To evaluate the antiproliferative activity of compound **XJ2‐2**, colony formation assays were conducted using EGFR mutant human NSCLC cell lines PC‐9 and H1975. The results indicated that **XJ2‐2** significantly inhibited the colony‐forming ability of both cell lines in a concentration‐dependent manner (Figure 5A). Furthermore, to assess the effects of **XJ2‐2** on tumor cell migration and invasion, wound healing assays and Transwell Matrigel invasion assays were performed. The wound healing assay revealed that **XJ2‐2** treatment significantly delayed the wound closure rate in PC‐9 and H1975 cells in a dose‐dependent manner, suggesting inhibition of their migratory capacity (Figure 5B). The Transwell invasion assay further confirmed that the number of cells invading through the Matrigel into the lower chamber was significantly reduced following 48 h of **XJ2‐2** treatment in a dose‐dependent manner (Figure 5C). In summary, these findings demonstrate that the small‐molecule compound **XJ2‐2** effectively suppresses the proliferation, migration, and invasive capacity of EGFR mutant NSCLC cell lines PC‐9 and H1975 in vitro.

### 2.7 Compound XJ2‐2 Induced Cancer Cell Apoptosis

**FIGURE 6:**
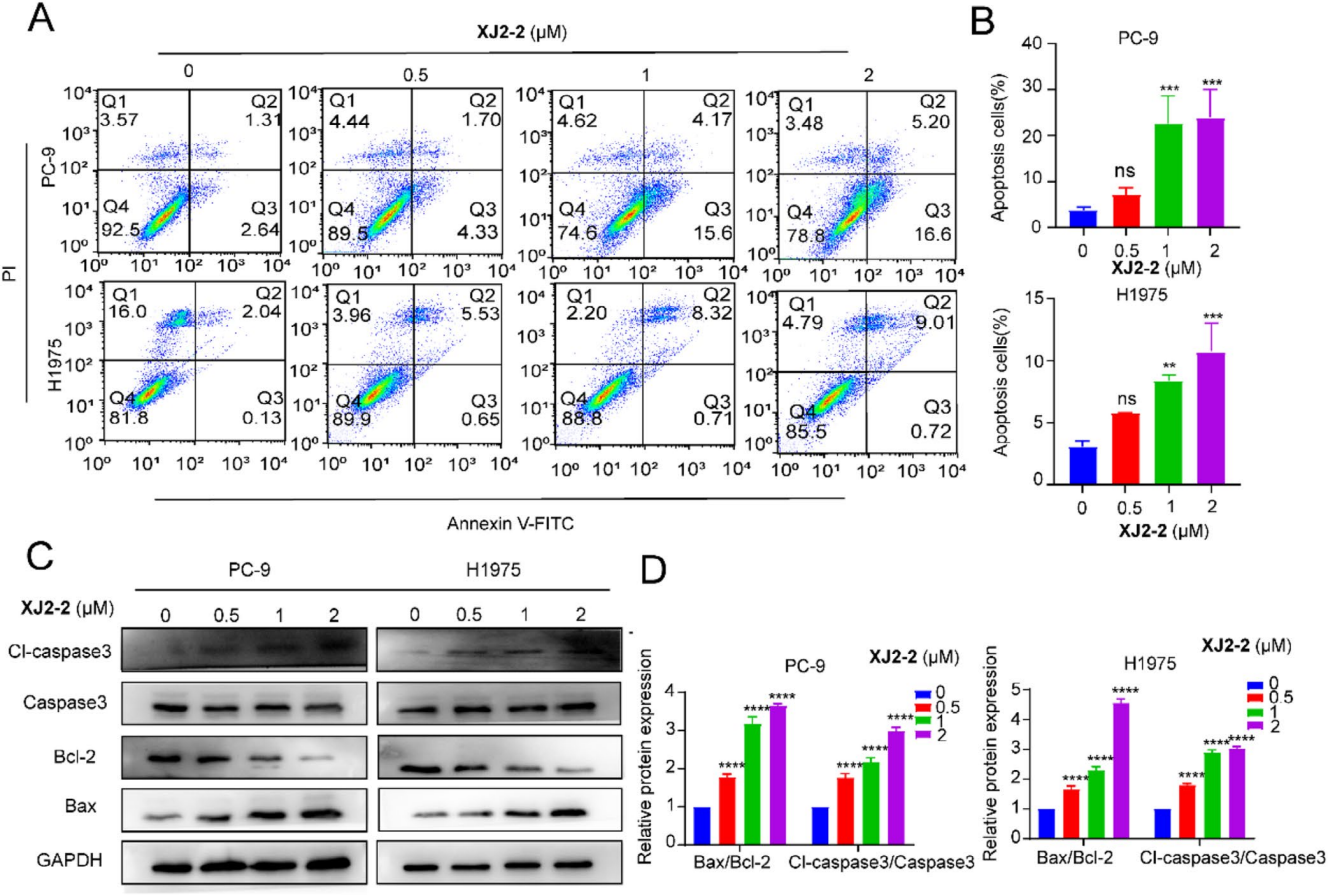
**XJ2‐2** induces concentration‐dependent apoptosis in NSCLC cells. (A, B) PC‐9 and H1975 cells were exposed to varying concentrations of **XJ2‐2** for 24 h, and the apoptotic ratios were quantified using flow cytometry. (C, D) Cells were treated with different concentrations of **XJ2‐2** for 24 h. Apoptosis‐related protein expression levels were assessed by Western blot analysis, normalized to GAPDH as a loading control, and band intensities were quantified using ImageJ software. Statistical significance was determined compared to the control group: *p < 0.05; **p < 0.01; ***p < 0.001; ****p < 0.0001.

To investigate whether the anti‐proliferative effects of **XJ2‐2** on EGFR‐mutant cell lines PC‐9 and H1975 are associated with apoptosis induction, we first quantified the apoptosis rates of PC‐9 and H1975 cells at various concentrations using Annexin‐V/PI staining. Flow cytometry analysis demonstrated that **XJ2‐2** significantly induced apoptosis in both PC‐9 and H1975 cells in a dose‐dependent manner (Figure 6A,B). Subsequently, we examined the expression levels of apoptotic proteins following **XJ2‐2** treatment via immunoblotting analysis. The results revealed an increase in Caspase 3 cleavage and the Bax/Bcl‐2 ratio (Figure 6C,D). Collectively, these findings indicate that **XJ2‐2** induces apoptosis in EGFR‐mutant cell lines PC‐9 and H1975.

### 2.8 Compound XJ2‐2 Induced Cell Cycle Arrest in Cancer Cells

**FIGURE 7:**
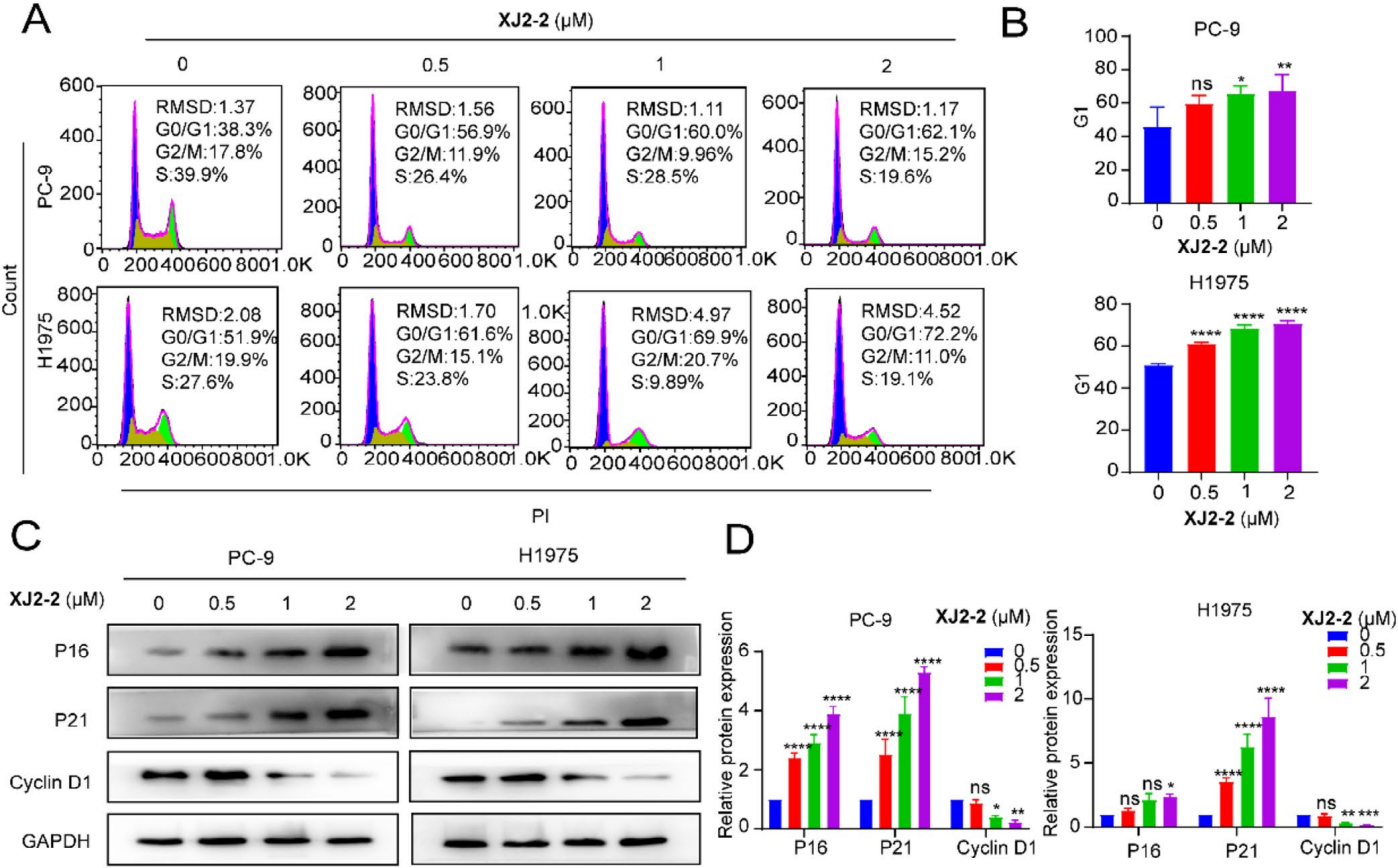
**XJ2‐2** induces G0/G1 phase arrest in NSCLC cells. (A, B) PC‐9 and H1975 cells were exposed to varying concentrations of **XJ2‐2** for 24 h, and the cell cycle distribution was subsequently analyzed by flow cytometry. (C, D) Cells were treated with the specified concentration of **XJ2‐2** for 24 h. Western blot analysis was performed to detect cell cycle‐related proteins, with GAPDH serving as a loading control. Band intensities were quantified using ImageJ software. Statistical significance compared to the control group is indicated as follows: *p < 0.05; **p < 0.01; ***p < 0.001; ****p < 0.0001.

To further elucidate the effects of **XJ2‐2** on cell viability and proliferation, we investigated its potential to induce cell cycle arrest. PC‐9 and H1975 cells were exposed to varying concentrations of **XJ2‐2** for 24 h, followed by propidium iodide (PI) staining to assess alterations in the G1, S, and G2/M phases of the cell cycle. Flow cytometry analysis revealed that as the concentration of **XJ2‐2** increased, the proportion of cells in the G1 phase significantly rose, while the proportions in the S and G2/M phases correspondingly decreased (Figure 7A,B). These findings indicate that **XJ2‐2** induces G1 phase cell cycle arrest in a dose‐dependent manner. Subsequently, WB was employed to evaluate the expression levels of proteins associated with the cell cycle following **XJ2‐2** treatment (Figure 7C,D). The results demonstrated that the expressions of P16 and P21 were upregulated in a dose‐dependent manner, whereas Cyclin D1 was downregulated in the same fashion. Collectively, these data suggest that **XJ2‐2** effectively suppresses the proliferative capacity of two EGFR‐mutant cell lines by inducing G1 phase arrest.

### 2.9 In Vivo Antitumor Potency and Mechanism of XJ2‐2

**FIGURE 8:**
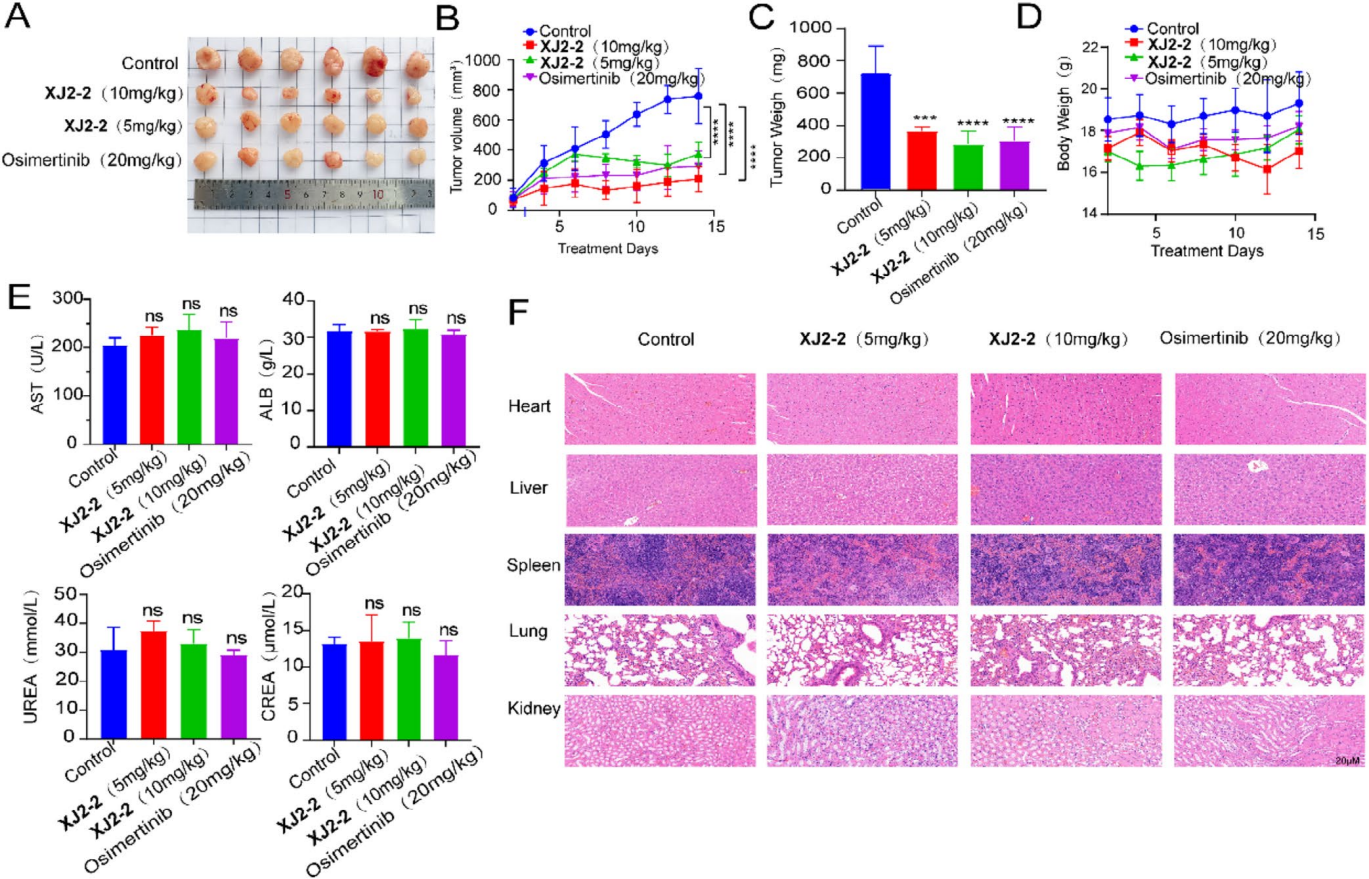
**XJ2‐2** inhibits the growth of non‐small cell lung cancer (NSCLC) tumors in vivo. (A) Representative images of tumors from different treatment groups. (B) Tumor volume changes over time. (C) Final tumor weights. (D) Changes in body weight of mice during treatment. (E) Serum biochemical parameters, including AST, ALB, UREA, and CREA, were analyzed from blood samples. (F) Hematoxylin and eosin (H&E) staining was performed on tissues from the heart, liver, spleen, lung, and kidney to evaluate potential organ toxicity. Data are presented as mean ± SD (*n* = 6). Statistical significance compared to the control group: *p < 0.05; **p < 0.01; ***p < 0.001; ****p < 0.0001.

Given that compound **XJ2‐2** exhibited potent antiproliferative activity and a well‐defined molecular mechanism of action in vitro, we further assessed its in vivo antitumor efficacy using a human tumor xenograft mouse model harboring the EGFR^DELL9^ mutation. The experimental protocol included an every‐other‐day intraperitoneal (i.p.) dosing schedule with two dose levels of **XJ2‐2** (5 mg/kg and 10 mg/kg), while osimertinib (20 mg/kg, p.o.) was administered as the positive control. The results demonstrated that, compared with the vehicle control group, **XJ2‐2** treatment significantly suppressed tumor growth. Notably, at the 10 mg/kg dose, the in vivo antitumor efficacy of **XJ2‐2** surpassed that of osimertinib. After 14 days of treatment, the mean tumor volume in the 10 mg/kg **XJ2‐2** group decreased to approximately 200 mm^3^, whereas the vehicle control group showed an increase to approximately 800 mm^3^. Compared with the osimertinib‐treated group (mean tumor volume ~300 mm^3^), the 10 mg/kg **XJ2‐2** group displayed significantly enhanced tumor growth inhibition, with a final mean tumor volume approximately 33% lower than that observed in the osimertinib group (Figure 8A–C). Moreover, continuous monitoring of body weight changes in the nude mice throughout the dosing period revealed no significant weight loss in either **XJ2‐2** treatment group across the tested dose range (5 and 10 mg/kg), indicating favorable tolerability and preclinical safety of the compound (Figure 8D).

To comprehensively evaluate the in vivo safety profile of compound **XJ2‐2**, histopathological analyses of the major organs (heart, liver, spleen, lung, and kidney) were performed in animals from each treatment group using hematoxylin and eosin (H&E) staining (Figure 8E,F). Concurrently, serum biochemical assays were carried out to monitor markers indicative of liver and kidney function and to identify potential toxic effects. The results revealed no significant pathological alterations in the H&E‐stained organ sections across all dose groups. Furthermore, all measured serum biochemical parameters remained within the normal physiological reference ranges. Collectively, the comprehensive toxicological assessment demonstrated that **XJ2‐2** exhibited a favorable safety profile at the tested doses, with no evidence of compound‐related toxicity or adverse effects.

**FIGURE 9:**
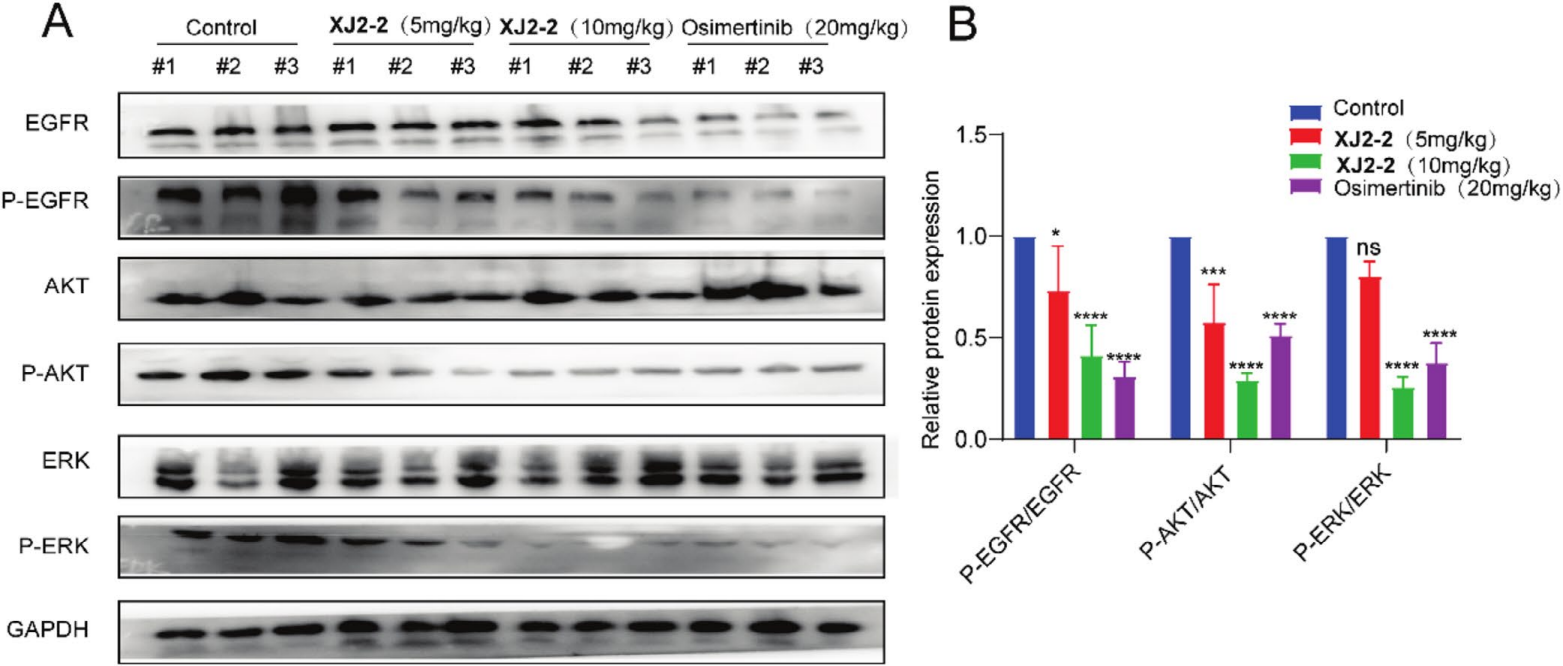
**XJ2‐2** inhibits the EGFR signaling pathway in vivo. (A) Proteins were extracted from tumor tissues, and the levels of P‐EGFR, P‐AKT, and P‐ERK were quantified by immunoblotting analysis. (B) Quantitative analysis of protein expression levels was performed. The representative data presented are derived from at least three independent experiments, including both control and drug‐treated groups (mean ± SD, *n* = 3). Statistical significance compared with the control group is indicated as follows: *p < 0.05, **p < 0.01, ***p < 0.001, and ****p < 0.0001.

Furthermore, to elucidate the mechanism of action of **XJ2‐2**, the effects of **XJ2‐2** on key proteins within the EGFR signaling pathway in tumor tissues were examined using WB analysis (Figure 9A,B). The results demonstrated that **XJ2‐2** (10 mg/kg) significantly inhibited the phosphorylation levels of EGFR, AKT, and ERK1/2, with an effect comparable to that of osimertinib (20 mg/kg). Both compounds markedly reduced the expression levels of P‐EGFR, P‐AKT, and P‐ERK1/2 in tumor tissues. This observation was consistent with the findings from previous in vitro cell experiments, further supporting the conclusion that **XJ2‐2** exerts its anti‐tumor activity through inhibition of the EGFR signaling pathway.

## 3 Conclusions

EGFR functions as a critical oncogenic driver in NSCLC, and drug resistance mediated by EGFR mutations represents a major clinical challenge in treatment. To address this issue, the present study implemented a structure‐based virtual screening approach to systematically evaluate a library of over 4000 compounds, ultimately identifying 4 novel candidate molecules with potential EGFR inhibitory activity. Among these, the compound **XJ2‐2** exhibited potent antiproliferative effects against NSCLC cell lines harboring common drug‐resistant EGFR mutations (PC‐9: EGFR^Del19^; H1975: EGFR^L858R/T790M^), with IC_50_ values of 0.88 ± 0.12 μM and 0.77 ± 0.09 μM, respectively. Mechanistic investigations confirmed that **XJ2‐2** selectively reduced the expression levels of mutant EGFR protein. Based on cellular thermal shift assay and molecular docking simulations, it was further demonstrated that **XJ2‐2** directly interacts with the EGFR kinase domain and effectively suppresses the phosphorylation of EGFR as well as its downstream signaling effectors, AKT and ERK1/2. This inhibition of the EGFR‐AKT‐ERK oncogenic signaling axis resulted in significant suppression of NSCLC cell proliferation, migration, and invasion, along with induction of apoptosis and G0/G1 phase cell cycle arrest. In the PC‐9 cell‐derived xenograft model, **XJ2‐2** administration markedly inhibited tumor growth, accompanied by decreased intratumoral levels of P‐EGFR, P‐AKT, and P‐ERK1/2. Comprehensive preclinical safety assessments confirmed the absence of treatment‐related adverse effects or significant toxicity across all tested dose levels. Collectively, **XJ2‐2**, as a novel molecular scaffold targeting EGFR drug‐resistant mutations, demonstrates strong potential for overcoming acquired resistance in NSCLC through effective modulation of the EGFR‐AKT‐ERK signaling pathway and represents a promising lead compound with substantial therapeutic development potential.

## 4 Experimental Methods and Materials

### 4.1 Docking‐Based Virtual Screening Virtual

Screening of drugs, a method that employs computational approaches to swiftly screen potential drug candidate molecules from a vast compound library, can significantly cut down experimental costs and shorten the experimental cycle. The core strategies of this method mainly consist of Structure‐Based Virtual Screening and Ligand‐Based Virtual Screening. Among them, Structure‐Based Virtual Screening depends on the three‐dimensional structural information of the target protein (such as X‐ray crystal structure, cryo‐electron microscopy structure, or homology modeling structure) to predict the binding mode and affinity between small molecules and the target.

In this research, the structure‐based virtual screening method was utilized to screen the compounds possessed by the research group. Specifically, the three‐dimensional atomic coordinates of EGFR (PDB ID: 2JIU) were retrieved from the Protein Data Bank (PDB; website: http://www.rcsb.org/). Molecular docking was performed using AutoDock Vina 1.2.3. Prior to docking, the receptor protein was processed in PyMol 2.5.2, including removal of water molecules, salt ions, and other small molecules. A docking box was then set up to fully enclose the entire protein. Additionally, all processed small molecules and the receptor protein were converted into the PDBQT format required by AutoDock Vina 1.2.3 using ADFRsuite 1.0. During docking, the exhaustiveness of the global search was set to 32, while all other parameters were kept at their default values. Immediately after that, the protonation state of the compound structure was set, and the 3D conformation was generated. Then, the Auto Dock Vina software was employed for molecular docking operations. Finally, 4 compounds (**XJ5‐24**, **XJ2‐2**, **XJ2‐7**, and **XJ5‐26**) were screened out. ased on a good G‐Score and the key interaction characteristics with the residues in the binding pocket. The synthetic route of compound **XJ2‐2** is outlined in Figure S1.

### 4.2 Molecule Dynamics

The initial structure for all‐atom molecular dynamics simulations was obtained from the small‐molecule–protein complex derived through docking, and simulations were performed using AMBER 24 software. Prior to simulation, the AM1‐BCC charges of the small molecule were calculated using the antechamber module. The small molecule and protein were then described by the GAFF2 force field and ff14SB force field, respectively. Hydrogen atoms were added to each system via the LEaP module, followed by the addition of a truncated octahedral TIP3P solvent box extending 10 Å beyond the solute. Na^+^/Cl^−^ ions were introduced to neutralize the system charge. Finally, topology and parameter files suitable for simulation were generated.

First, energy minimization was carried out using 2500 steps of steepest descent followed by 2500 steps of conjugate gradient optimization. After energy minimization, the system was gradually heated over 200 ps under constant volume and controlled heating rate, increasing the temperature slowly from 0 K to 298.15 K. Subsequently, a 500 ps NVT (isothermal‐isochoric) ensemble simulation was performed at 298.15 K to allow solvent molecules to distribute more uniformly within the solvent box. This was followed by a 500 ps NPT (isothermal‐isobaric) equilibration simulation. Finally, the entire system underwent a 100 ns NPT simulation under periodic boundary conditions. During the simulation, nonbonded interactions were truncated at 10 Å, long‐range electrostatics were treated using the Particle Mesh Ewald (PME) method, bond lengths involving hydrogen atoms were constrained using the SHAKE algorithm, and temperature control was achieved via the Langevin thermostat with a collision frequency γ set to 2 ps^−1^. The system pressure was maintained at 1 atm, with an integration time step of 2 fs, and trajectories were saved every 10 ps for subsequent analysis.

### 4.3 Cell Culture

Human normal lung epithelial cells (BESA‐2B, purchased from Suzhou Haixing Biotechnology Co. Ltd.) were cultured in Dulbecco's Modified Eagle Medium (DMEM) supplemented with 10% fetal bovine serum and 1% penicillin–streptomycin.

Human non‐small cell lung cancer cells (PC‐9 and H1975, purchased from Suzhou Haixing Biosciences Co. Ltd) were maintained in RPMI‐1640 medium containing 10% FBS and 1% penicillin–streptomycin. All cells were incubated at 37°C in a humidified atmosphere with 5% CO_2_.

### 4.4 Western Blotting

Cells or tissues were lysed in RIPA buffer supplemented with protease inhibitors (Sigma‐Aldrich). The protein concentration was quantified using the Pierce BCA Protein Assay Kit (Thermo Fisher Scientific, Waltham, MA, USA). Proteins were then separated by SDS‐PAGE and electrotransferred onto PVDF membranes (Merck Millipore, Burlington, MA, USA). The membranes were subsequently incubated with specific primary antibodies overnight at 4°C, followed by incubation with corresponding secondary antibodies at room temperature for 1 h. After washing three times with 1 × TBST, and exposed using the Tanon 5200 Imaging System (Shanghai, China).

### 4.5 Cell Activity Assays

PC‐9, H1975, and BESA‐2B cells were seeded into 96‐well cell culture plates at a density of 5000 cells per well and incubated in a humidified atmosphere containing 5% CO_2_ at 37°C for 24 h. Subsequently, the cells were treated with varying concentrations of the test compounds (0, 1, 2, 4, 8, 10 μM) or solvent control (vehicle). After a 72‐h treatment period, cell proliferation was assessed using the enhanced Cell Counting Kit‐8 (CCK‐8) from Elabscience Biotechnology Co. Ltd. (Wuhan, China). Specifically, 1/10 volume of CCK‐8 solution was added to each well and incubated at 37°C for 1–4 h. The absorbance (optical density, OD) at 450 nm was measured using a microplate reader (Multiskan GO 1510, ThermoFisher, USA). All experiments were performed in triplicate. Cell viability was calculated using the following formula: Cell viability (%) = [(OD_sample_ − OD_blank_)/(OD_control_ − OD_blank_)] × 100%.

### 4.6 Colony Formation Assay

PC‐9 and H1975 cells were seeded in 6‐well plates at a density of 1000 cells per well. The cells were subsequently treated with varying concentrations of the test compounds. Following a 15‐day culture period, the cells were fixed using 4% paraformaldehyde and stained with crystal violet. Cell images were then captured using a digital camera and quantitatively analyzed using ImageJ software. All experiments were independently repeated three times to ensure reproducibility.

### 4.7 Wound Healing Experiment

The impact of the compounds on cell migration was assessed using the wound healing assay. As previously described, cells were seeded in 6‐well plates at a density of 4 × 10^5^ cells per well and cultured under conditions of 5% CO_2_ at 37°C. Upon reaching 100% confluence, the monolayer was scratched with a pipette tip, and detached cells were removed by washing with PBS. Subsequently, the cells were exposed to designated concentrations of the compounds for 48 h. Images of the scratched areas were captured at 0 and 48 h using an inverted fluorescence microscope. The migration distance was quantified using ImageJ software. All experiments were performed in triplicate.

### 4.8 Transwell Assay for Cancer Cell Invasion

Cell invasion capability was assessed using a Matrigel‐coated transwell system. Specifically, each transwell chamber was coated with 80 μL of Matrigel diluted at a ratio of 1:8. Subsequently, 6 × 10^5^ cells were seeded into the upper chamber, and 100 μL of serum‐free RPMI‐1640 medium was added. Cells were then incubated with the designated concentration of **XJ2‐2** for 48 h. The lower chamber contained complete medium and was also incubated for 48 h. After incubation, non‐invading cells in the upper chamber were removed with a cotton swab. The remaining cells were fixed with 4% paraformaldehyde for 15 min and stained with crystal violet for 10 min. Microscopic images were captured, and the number of invasive cells was quantified using ImageJ software. All experiments were performed in triplicate to ensure reproducibility.

### 4.9 Flow Cytometry Analysis

The apoptosis of cells following **XJ2‐2** treatment was assessed using the Annexin V‐FITC/PI Apoptosis Detection Kit (Elabscience, Wuhan, China). Cells were exposed to varying concentrations of **XJ2‐2** (0, 0.5, 1, and 2 μM) for 48 h, subsequently digested with trypsin (EDTA‐free), and washed twice with cold phosphate‐buffered saline (PBS). They were then resuspended in 100 μL of 1× annexin V binding buffer. The cell suspension was incubated with 2.5 μL of annexin V‐FITC reagent and 2.5 μL of propidium iodide (PI) reagent in the dark at room temperature for 15–20 min. Following incubation, 400 μL of diluted 1× annexin V binding buffer was added and mixed thoroughly. Flow cytometric analysis was performed on the CytoFlex system (Beckman Coulter) within 1 h. For cell cycle analysis, cells treated with different concentrations of **XJ2‐2** (0, 0.5, 1, and 2 μM) for 48 h were fixed with 70% ethanol and stained with PI/RNase staining buffer. The cell cycle distribution was determined using the BD FACSCanto II flow cytometer. All experiments were conducted in triplicate.

### 4.10 Cell Thermal Stability Experiment

PC‐9 and H1975 cells were cultured in 10 cm culture dishes and subsequently treated with either DMSO or **XJ2‐2** for 2 h. Following treatment, the cells were harvested and resuspended in PBS within 10 PCR tubes. These tubes were then exposed to incremental heat treatments ranging from 38°C to 66°C at 4‐degree intervals, with each exposure lasting 3 min. After heat treatment, the samples were lysed and analyzed by Western blotting. All experiments were independently repeated three times to ensure reproducibility.

### 4.11 Animal Experiments

A total of 24 female Balb/C Nude mice (4–6 weeks old, 17–18 g) used in the experiment were provided by Beijing Huafukang Biotechnology Co. Ltd. (SCXK (Beijing) 2019‐008) and were housed at the Affiliated Tumor Hospital of Shandong First Medical University (SYXK (Lu) 20210012) for the animal experiments. The experimental animals were kept in an SPF environment with 45% humidity, constant temperature (23°C ± 2°C), and 12‐h light/dark cycles. Food and drinking water were routinely sterilized by autoclaving. Each mouse received a subcutaneous injection of 5 × 10^6^ PC‐9 cells in the armpit to establish a xenograft tumor model. Tumor volume was monitored every 2 days, and when tumors in all mice reached 50–100 mm^3^ (V = L × W^2^/2), the nude mice were randomly divided into 4 groups, with 6 mice in each group. The experiment included a control group, a low‐dose compound **XJ2‐2** group (5 mg/kg), a high‐dose compound **XJ2‐2** group (10 mg/kg), and a positive drug osimertinib group (20 mg/kg), with administration every other day for 14 days. During the treatment, the mice's body weight and tumor volume were measured every 2 days. In vivo toxicity assessment was conducted in mice anesthetized via intraperitoneal injection with ketamine (100 mg/kg) and xylazine (10 mg/kg). Blood samples were collected via retro‐orbital bleeding, centrifuged at 4000 rpm for 20 min, and analyzed using AST assay kit (Nanjing Jiancheng Bioengineering Institute, Catalog No. C010‐2‐1), ALT assay kit (Nanjing Jiancheng Bioengineering Institute, Catalog No. C009‐2‐1), and creatinine (CRE) assay kit (Nanjing Jiancheng Bioengineering Institute, Catalog No. C011‐2‐1). At the end of the treatment, all mice were euthanized and dissected. The obtained tumor tissues were weighed and recorded. Finally, tumor and other tissues were immediately frozen at −80°C or fixed in formalin solution for subsequent experiments. All animal experiments were conducted in accordance with the ARRIVE guidelines and were approved by the Animal Ethics Committee of Shandong Tumor Hospital (Approval No. SDTHEC2023006012).

### 4.12 Statistical Analysis

Significance was determined using GraphPad Prism Software 8.0. Data are represented as mean ± SD of triplicate experiments. Group differences were assessed using a Student's *t*‐test or one‐way ANOVA test. *p* < 0.05 was considered statistically significant. In the graphed data n.s., *, **, ***, **** denote *p* value of > 0.05, < 0.05, < 0.01, < 0.001 and < 0.0001.

## Author Contributions

**Mengfei Chen:** formal analysis, writing – review and editing. **Lijuan Zuo:** formal analysis, writing – review and editing. **Yuguo Liu:** funding acquisition, project administration, supervision, writing – review and editing. **Chuanfeng Liu:** supervision, project administration, writing – original draft, writing – review and editing. **Jing Shi:** funding acquisition, writing – review and editing, project administration, supervision. **Hanshuang Cai:** formal analysis, writing – review and editing. **Yuchen Zhao:** writing – original draft, methodology, formal analysis, data curation. **Ting Xu:** formal analysis, writing – original draft, methodology, data curation.

## Funding

This work was supported by Shandong Province Traditional Chinese Medicine Science and Technology Project (Z20243904), National Natural Science Foundation of China (22507064), and Shandong Provincial Natural Science Foundation (ZR2022MH243, ZR2023LZY008, ZR2025LSW009, and ZR2025LMB029).

## Conflicts of Interest

The authors declare no conflicts of interest.

## Supporting information


**Figure S1:** Synthetic route of compound **XJ2‐2**. Reagents and conditions: (i) K_2_CO_3_, DMF, 60°C, overnight; (ii) (ETO)_2_POCN, t‐BuOK, THF, 0°C to r.t.; (iii) Pd(OAc)_2_, BINAP, Cs_2_CO₃, 1,4‐dioxane, tert‐butyl 4‐(4‐aminophenyl)piperidine‐1‐carboxylate, reflux in N_2_ atmosphere; (iv) DCM, trifluoroacetic acid; (v) DCM, Et₃N, methanesulfonic anhydride.
**Figure S2:** The ^1^H NMR spectrum of compound **XJ2‐2**.
**Figure S3:** The MS spectrum of compound **XJ2‐2**.
**Figure S4:** Molecular docking results of the representative binding pose of compound **XJ2‐2** within the EGFR active site (PDB ID: 2JIU). The protein is shown as a surface representation, with key interacting residues labelled. Hydrogen bonds (blue dashed lines), π–*σ*, π–sulfur, and π–alkyl interactions are indicated.
**Figure S5:** Structural superposition of the docked ligand pose (light blue) onto the experimentally determined ligand conformation from the co‐crystal structure (pink). The calculated RMSD value is 1.714 Å, confirming the reliability of the docking protocol.
**Table S1:** Predicted binding energies of the top 10 docked conformations of compound **XJ2‐2**.

## Data Availability

The data that support the findings of this study are available on request from the corresponding author.

## References

[cbdd70391-bib-0001] Ahmad, I. , and H. M. Patel . 2025. “From Challenges to Solutions: A Review of Fourth‐Generation EGFR Tyrosine Kinase Inhibitors to Overcome the C797S Triple Mutat‐Ion in Non‐Small Cell Lung Cancer.” European Journal of Medicinal Chemistry 15, no. 284: 117178. 10.1016/j.ejmech.2024.117178 39724727

[cbdd70391-bib-0002] Avraham, R. , and Y. Yarden . 2011. “Feedback Regulation of EGFR Signalling: Decision Making by Early and Delayed Loops.” Nature Reviews. Molecular Cell Biology 12, no. 2: 104–117. 21252999 10.1038/nrm3048

[cbdd70391-bib-0003] Chen, L. , W. Fu , L. Zheng , Z. Liu , and G. Liang . 2018. “Recent Progress of Small‐Molecule Epidermal Growth Factor Receptor (EGFR) Inhibitors Against C797S Resistance in Non‐Small‐Cell Lung Cancer.” Journal of Medicinal Chemistry 61, no. 10: 4290–4300. 29136465 10.1021/acs.jmedchem.7b01310

[cbdd70391-bib-0004] Cho, B. C. , D. W. Kim , A. I. Spira , et al. 2023. “Amivantamab Plus Lazertinib in Osimertin‐Ib‐Relapsed EGFR‐Mutant Advanced Non‐Small Cell Lung Cancer: A Phase 1 Trial.” Nature Medicine 29, no. 10: 2577–2585. 10.1038/s41591-023-02554-7 PMC10579096 37710001

[cbdd70391-bib-0005] Cooper, A. J. , L. V. Sequist , and J. J. Lin . 2022. “Third‐Generation EGFR and ALK Inhibitors: Mechanisms of Resistance and Management.” Nature Reviews Clinical Oncology 19, no. 8: 499–514. 10.1038/s41571-022-00639-9. PMC9621058 35534623

[cbdd70391-bib-0006] DeSantis, C. E. , J. Ma , A. Goding Sauer , L. A. Newman , and A. Jemal . 2017. “Breast Cancer Statistics, 2017, Racial Disparity in Mortality by State.” CA: A Cancer Journal for Clinicians 67, no. 6: 439–448. 28972651 10.3322/caac.21412

[cbdd70391-bib-0007] Forli, S. , R. Huey , M. E. Pique , M. F. Sanner , D. S. Goodsell , and A. J. Olson . 2016. “Comput‐Ational Protein‐Ligand Docking and Virtual Drug Screening With the AutoDock‐ Suite.” Nature Protocols 11, no. 5: 905–919. 27077332 10.1038/nprot.2016.051 PMC4868550

[cbdd70391-bib-0008] Friesner, R. A. , J. L. Banks , R. B. Murphy , et al. 2004. “Glide: A New Approach for Rapid, Accurate Docking and Scoring. 1. Me‐Thod and Assessment of Docking Accuracy.” Journal of Medicinal Chemistry 47, no. 7: 1739–1749. 15027865 10.1021/jm0306430

[cbdd70391-bib-0009] Gao, C. , W. Wang , T. Liu , X. Li , Y. Yu , and J. Wu . 2025. “Annual Review of EGFR Inhi‐Bitors in 2024.” European Journal of Medicinal Chemistry 5, no. 292: 117677. 10.1016/j.ejmech.2025.117677 40328037

[cbdd70391-bib-0010] Gazdar, A. F. 2009. “Activating and Resistance Mutations of EGFR in Non‐Small‐Cell Lung Cancer: Role in Clinical Response to EGFR Tyrosine Kinase Inhibitors.” Oncogene 28, no. Suppl 1: S24–S31. 19680293 10.1038/onc.2009.198 PMC2849651

[cbdd70391-bib-0011] Halgren, T. A. , R. B. Murphy , R. A. Friesner , et al. 2004. “Glide: A New Approach for Rapid, Accurate Docking and Scoring. 2. Enrichment Factors in Database Screening.” Journal of Medicinal Chemistry 47, no. 7: 1750–1759. 15027866 10.1021/jm030644s

[cbdd70391-bib-0012] He, J. , Z. Zhou , X. Sun , et al. 2021. “The New Opportu‐Nities in Medicinal Chemistry of Fourth‐Generation EGFR Inhibitors to Over‐Come C797S Mutation.” European Journal of Medicinal Chemistry 15, no. 210: 112995. 10.1016/j.ejmech.2020.112995 33243531

[cbdd70391-bib-0013] Leiter, A. , R. R. Veluswamy , and J. P. Wisnivesky . 2023. “The Global Burden of Lung Cancer: Current Status and Future Trends.” Nature Reviews. Clinical Oncology 20, no. 9: 624–639. 10.1038/s41571-023-00798-3 37479810

[cbdd70391-bib-0014] Maemondo, M. , A. Inoue , K. Kobayashi , et al. 2010. “Gefitinib or Chemotherapy for Non‐Small‐Cell Lung Cancer With Mutated EGFR.” New England Journal of Medicine 362, no. 25: 2380–2388. 20573926 10.1056/NEJMoa0909530

[cbdd70391-bib-0015] Meng, X. Y. , H. X. Zhang , M. Mezei , and M. Cui . 2011. “Molecular Docking: A Powerful Approach for Structure‐Based Drug Discovery.” Current Computer‐Aided Drug Design 7, no. 2: 146–157. 21534921 10.2174/157340911795677602 PMC3151162

[cbdd70391-bib-0016] Mok, T. S. , Y.‐L. Wu , M.‐J. Ahn , et al. 2017. “Osimertinib or Platinum‐Pemetrexed in EGFR T790M‐Pos‐Itive Lung Cancer.” New England Journal of Medicine 376, no. 7: 629–640. 27959700 10.1056/NEJMoa1612674 PMC6762027

[cbdd70391-bib-0017] Niggenaber, J. , J. Hardick , J. Lategahn , and D. Rauh . 2020. “Structure Defines Function: Clinically Relevant Mutations in ErbB Kinases.” Journal of Medicinal Chemistry 63, no. 1: 40–51. 31414802 10.1021/acs.jmedchem.9b00964

[cbdd70391-bib-0018] Patel, H. , R. Pawara , A. Ansari , and S. Surana . 2017. “Recent Updates on Third Generatio‐n EGFR Inhibitors and Emergence of Fourth Generation EGFR Inhibitors to Combat C797S Resistance.” European Journal of Medicinal Chemistry 142: 32–47. 28526474 10.1016/j.ejmech.2017.05.027

[cbdd70391-bib-0019] Paz‐Ares, L. , E. H. Tan , K. O'Byrne , et al. 2017. “Afatinib Versus Gefitinib in Patients With EGFR Mutation‐Positive Advanced Non‐Small‐Cell Lung Cancer: Overall Survival Data From the Phase IIb LUX‐Lung 7 Trial.” Annals of Oncology 28, no. 2: 270–277. 28426106 10.1093/annonc/mdw611 PMC5391700

[cbdd70391-bib-0020] Piotrowska, Z. , H. Isozaki , J. K. Lennerz , et al. 2018. “Landscape of Acquired Resistance to Osimertinib in *EGFR*‐Mutant NSCLC and Clinical Validation of Combined EGFR and RET Inhibition With Osimertinib and BLU‐667 for Acquired *RET* Fusion.” Cancer Discovery 8, no. 12: 1529–1539. 30257958 10.1158/2159-8290.CD-18-1022 PMC6279502

[cbdd70391-bib-0021] Remon, J. , C. E. Steuer , S. S. Ramalingam , and E. Felip . 2018. “Osimertinib and Other Third‐Generation EGFR TKI in EGFR‐Mutant NSCLC Patients.” Annals of Oncology 29, no. suppl_1: i20–i27. 29462255 10.1093/annonc/mdx704

[cbdd70391-bib-0022] Rosell, R. , T. Morán , E. Carcereny , et al. 2010. “Non‐Small‐Cell Lung Cancer Harbouring Mutations in the EGFR Kinase Domain.” Clinical and Translational Oncology 12, no. 2: 75–80. 20156777 10.1007/S12094-010-0473-0

[cbdd70391-bib-0023] Sabe, V. T. , T. Ntombela , L. A. Jhamba , et al. 2021. “Current Trends in Computer Aided Drug Design and a Highlight of Drugs Discovered via Computational Techniques: A Review.” European Journal of Medicinal Chemistry 15, no. 224: 113705. 10.1016/j.ejmech.2021.113705 34303871

[cbdd70391-bib-0024] Sharma, S. V. , D. W. Bell , J. Settleman , and D. A. Haber . 2007. “Epidermal Growth Factor Re‐Ceptor Mutations in Lung Cancer.” Nature Reviews. Cancer 7, no. 3: 169–181. 17318210 10.1038/nrc2088

[cbdd70391-bib-0025] Shepherd, F. A. , J. Rodrigues Pereira , T. Ciuleanu , et al. 2005. “Erlotinib in Previously Treated Non‐Small‐Cell Lung Cancer.” New England Journal of Medicine 353, no. 2: 123–132. 16014882 10.1056/NEJMoa050753

[cbdd70391-bib-0026] Tan, C. S. , N. B. Kumarakulasinghe , Y. Q. Huang , et al. 2018. “Third Generation EGFR TKIs: Current Data and Future Directions.” Molecular Cancer 17, no. 1: 29. 29455654 10.1186/s12943-018-0778-0 PMC5817792

[cbdd70391-bib-0027] Thress, K. S. , C. P. Paweletz , E. Felip , et al. 2015. “Acquired EGFR C797S Mutation Mediates Resistance to AZD9291 in Non‐Small Cell Lung Cancer Harboring EGFR T790M.” Nature Medicine 21, no. 6: 560–562. 10.1038/nm.3854 PMC4771182 25939061

[cbdd70391-bib-0028] Tsuboi, M. , R. S. Herbst , T. John , et al. 2023. “Overall Survival With Osimertinib in Resected *EGFR*‐Mutated NSCLC.” New England Journal of Medicine 389, no. 2: 137–147. 37272535 10.1056/NEJMoa2304594

[cbdd70391-bib-0029] Westover, D. , J. Zugazagoitia , B. C. Cho , C. M. Lovly , and L. Paz‐Ares . 2018. “Mechanisms of Acquired Resistance to First‐ and Second‐Generation EGFR Tyrosine Kina‐Se Inhibitors.” Annals of Oncology 29, no. suppl_1: i10–i19. 29462254 10.1093/annonc/mdx703 PMC6454547

[cbdd70391-bib-0030] Wheeler, D. L. , E. F. Dunn , and P. M. Harari . 2010. “Understanding Resistance to EGFR Inhi‐Bitors‐Impact on Future Treatment Strategies.” Nature Reviews. Clinical Oncology 7, no. 9: 493–507. 10.1038/nrclinonc.2010.97 PMC2929287 20551942

[cbdd70391-bib-0031] Woo, H. M. , X. Qian , L. Tan , et al. 2023. “Optimal Decision‐Making in High‐Throughput Virtual Screening Pipelines.” Pat‐Terns 4, no. 11: 100875. 10.1016/j.patter.2023.100875 PMC10682755 38035191

[cbdd70391-bib-0032] Yun, C. H. , K. E. Mengwasser , A. V. Toms , et al. 2008. “The T790M Mutation in EGFR Kinase Causes Drug Res‐Istance by Increasing the Affinity for ATP.” Proceedings of the National Academy of Sciences of the United States of America 105, no. 6: 2070–2075. 18227510 10.1073/pnas.0709662105 PMC2538882

