# Supplementary material for: Discovery of Potent EGFR Inhibitors for Del19 and L858R/T790M Mutants Through Structure‐Based Virtual Screening and Biological Evaluation
Source: Chem Biol Drug Des. 2026 Sep 11;108(3):e70391. doi: 10.1111/cbdd.70391 (PMC13563102; doi:10.1111/cbdd.70391)
Supplement: Supplementary file 1 [file CBDD-108-e70391-s001.docx]

**Discovery of Potent EGFR Inhibitors for Del19 and L858R/T790M Mutants through Structure-Based Virtual Screening and Biological Evaluation**

Ting Xu ^a,#^, Yuchen Zhao ^a,#^, Mengfei Chen ^a^, Lijuan Zuo ^b^, Hanshuang Cai ^a^, Yuguo Liu ^a,*^, Chuanfeng Liu ^a,*^, and Jing Shi ^a,*^

***^a^*** *Department of Pharmacy (Shandong Provincial Key Traditional Chinese Medical Discipline of Clinical Chinese Pharmacy), Shandong Cancer Hospital and institute, Shandong First Medical University and Shandong Academy of Medical Sciences, Jinan 250117, PR China*

***^b^*** *Taian Central Hospital General ICU Zone 2, No. 29 Longtan Road, Taian City 271000, China*

***^#^ These authors contributed equally.***

****Corresponding authors.*** *E-mail address:* [*ygliu@sdfmu.edu.cn*](mailto:ygliu@sdfmu.edu.cn) *(Yuguo Liu),* [*liuchuanfeng214@163.com (Chuanfeng Liu),*](rewritten://fb5635ee-0f94-40ef-b70a-0b009873ef02) *shijing@sdfmu.edu.cn (Jing Shi)*

**Tables of Contents**

**1. The Synthesis of Compound XJ2‑2**

**Figure S1: Synthetic route of compound XJ2‑2.**

**2. NMR Spectra of Compound XJ2‑2**

**Figure S2: The ^1^H NMR spectrum of compound XJ2-2**

**Figure S3: The MS spectrum of compound XJ2‑2.**

**3. Redocking results**

**Table S1: Binding energy of docking conformations.**

**Figure S4: Molecular docking results of XJ2‑2 with EGFR.**

**Figure S5: Structural superposition of the docked ligand pose onto the experimentally determined ligand conformation from the co‑crystal structure.**

**4. References**

**1.The Synthesis of Compound XJ2-2**

**1.1 General.** All chemical reagents and solvents were procured from commercial suppliers with a purity of ≥ 97%. Thin-layer chromatography (TLC) was carried out on Merck silica gel GF254 plates, and the product spots were visualized under 254 nm UV light. Flash column chromatography was performed using silica gel (200–300 mesh) supplied by Haiyang Chemical Company, Qingdao. The melting points of the compounds were measured using a Tianjin Tian Guang Optical Instrument Factory RY-1G micro-melting point apparatus (uncorrected). ¹H NMR spectra were recorded on a Bruker AV-400 spectrometer, with tetramethylsilane (TMS) as the internal standard and deuterated dimethyl sulfoxide (DMSO-*d₆*) as the solvent; chemical shifts are reported in ppm and coupling constants (J) in Hz. Mass spectrometry (MS) data were acquired on an API 4000 LC/MS spectrometer from Applied Biosystems, USA.

**1.2** **The synthetic route of the compound XJ2-2**

**Figure S1: Synthetic route of compound XJ2‑2.** (ⅰ) K_2_CO_3_, DMF, 60℃,overnight; (ⅱ) (ETO)_2_POCN, t-BuOK, THF, 0℃ to r.t.; (ⅲ) Pd(OAc)_2_, BINAP, Cs_2_CO_3_, 1,4-dioxane, tert-butyl 4-(4-aminophenyl)piperidine-1-carboxylate, reflux in N_2_ atmosphere; (ⅳ) DCM, Trifluoroacetic acid; (ⅴ) DCM, Et_3_N, methanesulfonic anhydride.

**The synthesis of compound X-2 was conducted as follows^[1]^**: 2,4-Dibromopyrimidine (20 mg, 0.084 mmol), 4-hydroxy-3,5-dimethylbenzaldehyde (13.9 mg, 0.092 mmol), and anhydrous K_2_CO_3_ (12.7 mg, 0.092 mmol) were dissolved in anhydrous N,N-dimethylformamide (1 mL). The reaction mixture was stirred at room temperature for 12 hours. The progress of the reaction was monitored by thin-layer chromatography (TLC) until complete consumption of 2,4-dibromopyrimidine was confirmed. Upon completion, the reaction mixture was carefully poured into ice-cooled water (5 mL) to quench the reaction. The pH of the aqueous phase was adjusted to neutrality using 12 M hydrochloric acid. The aqueous layer was then extracted with dichloromethane (DCM, 3 × 5 mL). The organic layers were combined, washed with saturated NaCl solution, and dried over anhydrous Na_2_SO_4_. The drying agent was subsequently removed by filtration, and the filtrate was concentrated under reduced pressure. The crude residue was purified by silica gel column chromatography using petroleum ether/ethyl acetate (30:1, v/v) as the eluent, yielding compound **X-2** as a white solid in 86.6% yield (22.65 mg).

**The synthesis of compound X-3 was carried out as follows**: Under an argon atmosphere at 0 °C, diethyl cyano(methyl)phosphonate (15.2mL, 97.5 mmol) and potassium tert-butoxide (10.94g, 97.5 mmol) were dissolved in anhydrous tetrahydrofuran (1 mL) and stirred for 30 minutes to allow for activation. Subsequently, compound **X-2** (20 mg, 0.065 mmol) was added, and the ice bath was removed. The reaction mixture was then allowed to stir at room temperature for 12 hours. The progress of the reaction was monitored by TLC until complete consumption of compound **X-2** was confirmed. Upon completion, the reaction was quenched by the addition of water (5 mL). The resulting mixture was extracted with DCM (3 × 5 mL). The organic layers were combined, washed sequentially with saturated sodium chloride solution, and dried over anhydrous sodium sulfate. After filtration to remove the drying agent, the solvent was evaporated under reduced pressure. The crude residue was purified by silica gel column chromatography using petroleum ether/ethyl acetate (15:1, v/v) as the eluent, affording compound **X-3** as a pale yellow solid in 84.9% yield (18.17 mg).

**The synthesis of compound X-4 was carried out under a nitrogen atmosphere as follows^[2]^**: Compound **X-3** (20 mg, 0.06 mmol), Pd(OAc)₂ (1.34 mg, 0.006 mmol, 10 mol%), 4,5-bis(diphenylphosphino)-9,9-dimethylxanthene (4 mg, 0.006 mmol, 10 mol%), and Cs₂CO₃ (39 mg, 0.12 mmol, 2.0 equiv) were dissolved in anhydrous 1,4-dioxane (2 mL). Subsequently, tert-butyl 4-(4-aminophenyl)piperidine-1-carboxylate (33.1 mg, 0.12 mmol, 2.0 equiv) was added to the reaction mixture. The resulting solution was stirred at room temperature for 12 hours. The progress of the reaction was monitored by TLC until complete consumption of compound **X-3** was confirmed. Upon completion, the reaction was quenched by the addition of water (5 mL). The mixture was extracted with a DCM/EtOAc (1:1, v/v; 3 × 5 mL). The organic layers were combined, washed with saturated NaCl solution, and dried over anhydrous Na_2_SO_4_. The drying agent was removed by filtration, and the filtrate was concentrated under reduced pressure. The crude residue was purified by silica gel column chromatography using petroleum ether/ethyl acetate (15:1, v/v) as the eluent, yielding compound **X-4** as a white solid in 44.2% yield (15.9 mg).

**The synthesis of compound X-5 (Boc deprotection) was performed as follows**: Compound **X-4** (20 mg, 0.06 mmol) was dissolved in anhydrous dichloromethane (2 mL), and trifluoroacetic acid (TFA, 68 μL, 0.88 mmol, 5.0 equiv) was added dropwise at 0 °C. Following completion of the addition, the reaction mixture was stirred at 0 °C for 30 minutes. The progress of the reaction was monitored by TLC until complete consumption of compound **X-4** was confirmed. Upon completion, the reaction was quenched by the addition of ice-cooled water (5 mL), and the pH of the aqueous phase was carefully adjusted to 8–9 using saturated K_2_CO_3_ solution. The aqueous layer was extracted with DCM (3 × 5 mL). The organic layers were combined, washed with saturated NaCl solution, and dried over anhydrous Na_2_SO_4_. The drying agent was removed by filtration, and the filtrate was concentrated under reduced pressure. The crude residue was purified by silica gel column chromatography using petroleum ether/ethyl acetate (15:1, v/v) as the eluent, yielding compound **X-5** as a white solid in 55.6% yield (11.1 mg).

**The synthesis of compound XJ2-2 was carried out as follows^[2]^:** Compound **X-5** (20 mg, 0.047 mmol) and methanesulfonyl chloride (72.6 mg, 0.14 mmol, 3.0 equiv) were dissolved in anhydrous DCM (2 mL), and Et₃N (19.5 μL, 0.14 mmol, 3.0 equiv) was added dropwise at 0 °C. Following completion of the addition, the reaction mixture was stirred at room temperature for 12 hours. The progress of the reaction was monitored by TLC until complete consumption of compound **X-5** was confirmed. Upon completion, the reaction was quenched by the addition of ice-cooled water (5 mL), and the aqueous layer was extracted with a DCM/EtOAc (1:1, v/v; 3 × 5 mL). The organic layers were combined, washed with saturated sodium chloride solution, and dried over anhydrous Na_2_SO_4_. The drying agent was removed by filtration, and the filtrate was concentrated under reduced pressure. The crude residue was purified by silica gel column chromatography using petroleum ether/ethyl acetate (15:1, v/v) as the eluent, yielding compound **XJ2-2** as a white solid in 44.2% yield (10.6 mg). ^1^H NMR (800 MHz, DMSO) δ 9.52 (s, 1H), 8.36 (d, *J* = 5.5 Hz, 1H), 7.69 – 7.66 (m, 1H), 7.53 (s, 2H), 7.28 (s, 2H), 6.91 (s, 3H), 6.49 (d, *J* = 3.4 Hz, 1H), 3.66 (d, *J* = 11.8 Hz, 2H), 3.36 (s, 1H), 2.80 (s, 3H), 2.77 (d, *J* = 12.1 Hz, 2H), 2.09 (s, 6H), 1.80 (d, *J* = 15.0 Hz, 2H), 1.61 – 1.55 (m, 2H). ESI-MS: m/z 504.2069 (M + H)^+^. C_27_H_29_N_5_O_3_S [503.1991].

**2.The ^1^H NMR and MS spectrum of compound XJ2-2**

**Figure S2:** **The ^1^H NMR spectrum of compound XJ2-2.**

**Figure S3: The MS spectrum of compound XJ2-2.**

**3.Redocking results**

We ranked the top 10 docked conformations by docking score and selected the lowest-binding-energy conformation—evaluated using estimated binding free energy (Table S1)—as the representative binding pose for detailed interaction analysis (Figure S4). As illustrated in Figure 1, the protein engages the small-molecule ligand through multiple noncovalent interactions, including hydrogen bonds, π–σ, π–sulfur, and π–alkyl contacts. Superimposition of this pose onto the experimentally determined ligand conformation from the crystal structure yielded an RMSD of 1.714 Å (Figure S5), confirming the protocol’s ability to reliably reproduce native binding geometry and thereby supporting its robustness for downstream virtual screening and structure-guided drug design.

**Table S1: Binding energy of docking conformations.**

| Rank | Binding Energy (kcal/mol) |
| --- | --- |
| 1 | -9.7 |
| 2 | -9.2 |
| 3 | -9.0 |
| 4 | -8.6 |
| 5 | -8.6 |
| 6 | -8.5 |
| 7 | -8.5 |
| 8 | -8.0 |
| 9 | -7.7 |
| 10 | -7.4 |

**Figure S4: Molecular docking results.** Molecular docking results of the representative binding pose of compound XJ2-2 within the EGFR active site (PDB ID: 2JIU). The protein is shown as a surface representation, with key interacting residues labelled. Hydrogen bonds (blue dashed lines), π–σ, π–sulfur, and π–alkyl interactions are indicated.

**Figure S5: Structural superposition of the docked ligand pose (light blue) onto the experimentally determined ligand conformation from the co-crystal structure (pink).**

**4.References**

[1] Dai J, Jiang X, Gao H, Huang B, De Clercq E, Pannecouque C, Du S, Liu X, Zhan P. Discovery of novel fused-heterocycle-bearing diarypyrimidine derivatives as HIV-1 potent NNRTIs targeting tolerant region I for enhanced antiviral activity and resistance profile. Eur J Med Chem. 2025; 281:117033.
[2] Jiang X, Huang B, Rumrill S, Pople D, Zalloum WA, Kang D, Zhao F, Ji X, Gao Z, Hu L, Wang Z, Xie M, De Clercq E, Ruiz FX, Arnold E, Pannecouque C, Liu X, Zhan P. Discovery of diarylpyrimidine derivatives bearing piperazine sulfonyl as potent HIV-1 nonnucleoside reverse transcriptase inhibitors. Commun Chem. 2023; 6(1):83.
